# Indigenous knowledge and plant species used as mosquito repellents in the West Nile Subregion, Uganda

**DOI:** 10.1186/s41182-025-00831-4

**Published:** 2025-11-07

**Authors:** Benson Oloya, Morgan Andama, Betty Akwongo, Paulino Amagu, Robert Opoke, Milton Candia, Rehemah Samanya, Philliam Taban, Emoses Agen Okello, Godwin Anywar

**Affiliations:** 1 Department of Chemistry, Faculty of Science, Muni University, P.O. Box 725, Arua, Uganda; 2 Department of Biology, Faculty of Science, Muni University, P.O. Box 725, Arua, Uganda; 3 Department of Nursing, Faculty of Health Sciences, Muni University, P.O. Box 725, Arua, Uganda; 4 Department of Physics, Faculty of Science, Muni University, P.O. Box 725, Arua, Uganda; 5 Department of Plant Sciences, Microbiology and Biotechnology, College of Natural Sciences, Makerere University, P.O. Box 7062, Kampala, Uganda; 6 Ethnopharmacology and Zoopharmacognosy, Bernhard Nocht Institute for Tropical Medicine, Hamburg, Germany; 7 Department of Cellular Biophysics, Max Planck Institute for Medical Research, Bildungscampus Heilbronn, Jahnstrasse 29, 69120 Heidelberg, Germany

**Keywords:** Malaria, Medicinal plants, Ethnobotanical, Traditional medicine, Indigenous knowledge, Mosquito repellents

## Abstract

**Background:**

Female *Anopheles* mosquitoes are the primary vectors for malaria transmission within communities, significantly contributing to the high burden of malaria in Africa overall and Uganda specifically. Many tropical plants have insect-repellent properties and have traditionally been used in their native regions to prevent mosquito bites.

**Methods:**

A cross-sectional ethnobotanical survey was conducted between January 2025 and May 2025 in five districts of the West Nile Subregion: Adjumani, Moyo, Madi-Okollo, Pakwach, and Obongi. Semi-structured questionnaires were used to collect data on indigenous knowledge about mosquito repellents from 57 respondents, who were selected through purposive and snowball sampling techniques. The ethnobotanical data were analyzed using descriptive statistics, the informant consensus factor, and preference ranking.

**Results:**

A total of 42 plant species from 40 genera and 25 families were documented as being used to repel mosquitoes. The plants most commonly used for this purpose were: *Azadirachta indica *A.Juss. (9)*, Boswellia papyrifera* (Caill.) (7), *Aeschynomene american*a L. (6), *Mesosphaerum suaveolens* (L.) Kuntze. (6), and *Ocimum gratissimum* L. (5). The most common indigenous method for repelling mosquitoes involves burning either dry cow dung (32%) or dry goat droppings (20%). Most of the plant species belong to the families Fabaceae (10), Lamiaceae (4), and Asteraceae (3). The majority of the plant species used were trees (43%) and herbs (42%), with leaves (42%) and seeds (12%) being the most frequently used plant parts. Except for *Cymbopogon citratus* (DC.) Stapf and *M. suaveolens,* which are used as live plants for repelling mosquitoes, all other plant species used were prepared by burning or smoking indoors.

**Conclusions:**

Communities in the West Nile Subregion, especially those living along the River Nile, possess rich indigenous knowledge and practices used to repel mosquitoes in their efforts to control deadly malaria.

## Background

Globally, Africa bears the heaviest burden of malaria, with 94% of cases reported in 2023 [1]. Moreover, as of 2023, Uganda ranked third in the world in terms of malaria cases, accounting for 4.8% of the cases [1]. Mosquitoes, especially female *Anopheles* species, are the primary vectors for transmitting malaria within humans [2]. During blood feeding, an infected mosquito introduces *Plasmodium* parasites into the host’s bloodstream through its saliva, initiating the infection process [3]. The mosquito bites cause discomfort, skin irritation, and rashes, in addition to spreading the malaria parasite [4]. This poses a threat as a potential source of malaria parasites that can be spread to cause malaria epidemics in the population if mosquitoes continue to bite uncontrollably [5].

In the West Nile Subregion, malaria accounts for 62% of outpatient care for children under five years of age and pregnant women in health facilities, 35% of inpatient admissions, and 4% of deaths among children under 5 years of age and pregnant women [6]. These figures indicate a high rate of malaria infections in the Subregion.

Worse still, global warming caused by climate change is expanding the geographic range of mosquito habitats, thereby facilitating the spread of vector-borne diseases. The expected spread of malaria to higher altitudes and temperate regions indicates a risk of outbreaks in populations with little to no prior immunity and where public health infrastructure may be inadequately prepared to respond. As these risks increase, strengthened prevention and control strategies are urgently needed [7–9].

The use of modern medicine is considered the gold standard in the treatment of malaria. However, the widespread reliance on these contemporary drugs has contributed to the emergence and spread of drug resistance, particularly for frontline therapies such as artemisinin, which threatens long-term treatment efficacy [10–12]. Moreover, many antimalarial medicines can cause adverse effects, including gastrointestinal disturbances, neurotoxicity, and, in rare cases, severe allergic reactions, which can affect patient compliance [13–15]. In addition, modern antimalarials are also often expensive and may not be accessible to populations in remote or resource-limited areas, resulting in delayed treatment and a greater disease burden [16, 17]. Furthermore, the pharmaceutical approach mainly targets the parasite without addressing the broader social and environmental factors that facilitate malaria transmission, such as poor housing and stagnant water [18].

Traditionally, malaria prevention involves avoiding mosquito bites and using various methods and materials, including plants with mosquito-repelling properties [19, 20]. Many plants with insect-repelling properties are native to the tropics and have been used for a wide range of medicinal purposes, including a history of use for personal protection against biting insects [21]. However, there are no scientific reports on plants and other materials traditionally used to repel mosquitoes in the West Nile Subregion. The indigenous knowledge about medicinal plants used as mosquito repellents is still passed down orally, which risks being lost across generations. The aim of this study was therefore to investigate the use of plants and other materials in the prevention of mosquito bites in the West Nile region.

## Methods

### Description of the study area

**Fig. 1:**
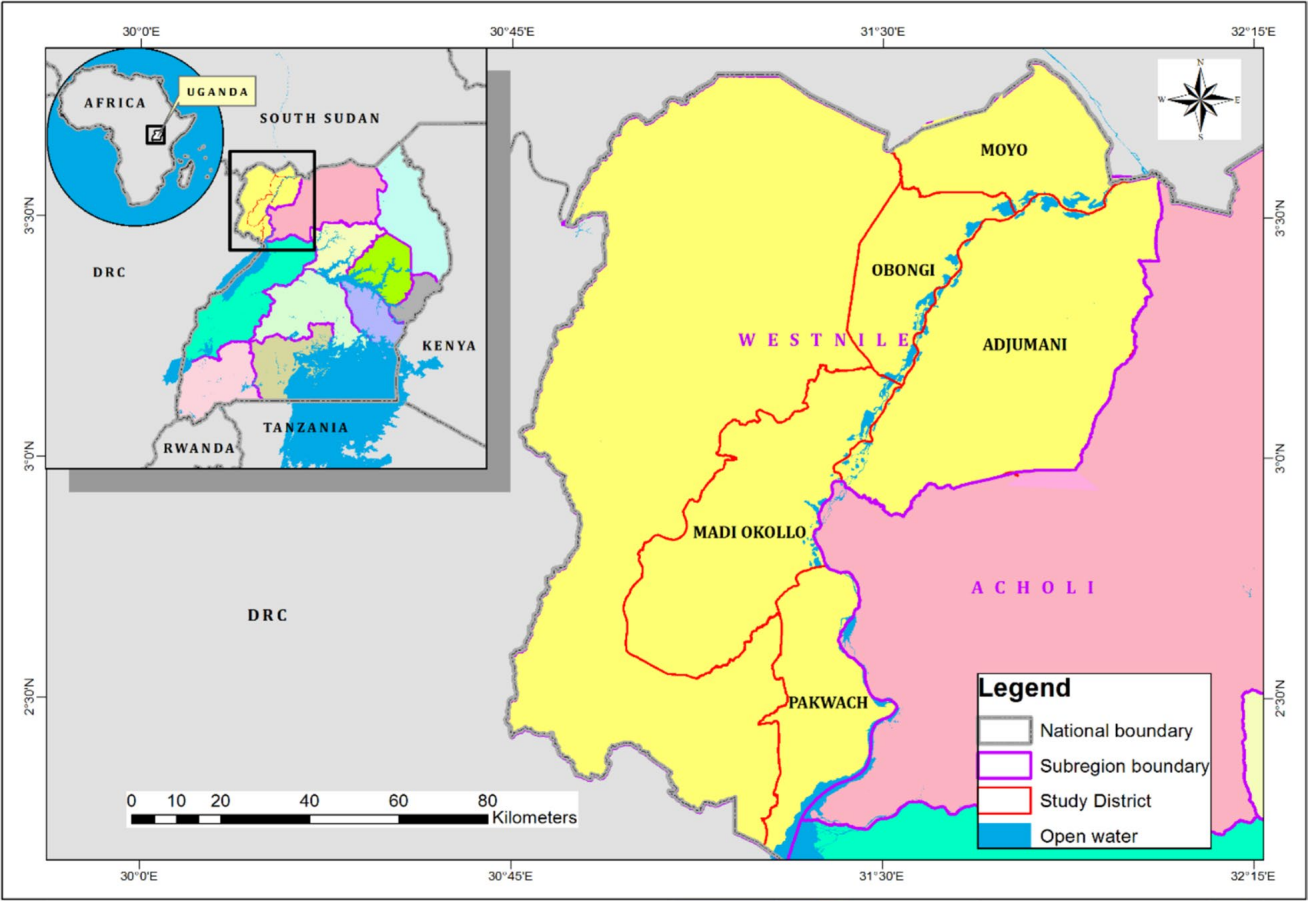
Map of the study area in the West Nile Subregion

The study was conducted in 5 out of the 12 districts of the West Nile Subregion in Uganda (Fig. 1). The subregion is located in the north-western part of the country with the coordinates Lat: 2.20°N to 3.85°N and Long: 30.60°E to 32.00°E [22]. These five districts, namely Adjumani, Moyo, Madi-Okollo, Pakwach, and Obongi, were chosen because they are found along the River Nile, whose extensive network of swamps and tributaries makes them ideal breeding grounds for mosquitoes [23, 24].

On the other hand, these districts are well endowed with plant diversity because the subregion receives an average annual rainfall of 1259 mm [22], which supports the growth of various plant species, some of which are used as mosquito repellents and for the treatment of malaria [25]. Unfortunately, this area is also under intense pressure from several activities, such as land conversion for agriculture and refugee settlements, which are causing the loss of natural biodiversity, including plant species with potential mosquito repellent properties and other uses [22].

The subregion is inhabited mainly by the Lugbara, Madi, Alur, Kakwa, Aringa, Lendu, Jonam and Kebu tribes, each with diverse indigenous traditional practices for controlling malaria. Therefore, it is important to document their unique and even similar attributes.

### Research design

A descriptive cross-sectional study employing a quantitative research approach was conducted.

### Sampling techniques

Purposive sampling was used to select study participants who were traditional medicine practitioners (TMPs) [26], with help from local leaders (local councils). Then, snowball sampling [27] was utilized to find additional participants on the basis of their skills, knowledge, and experience in medicinal plant use, as recommended by the initial participants.

### Study population

The study subjects included TMPs, who were seen as custodians of traditional medicine practices in their communities. Moreover, the TMPs were considered qualified if they possessed extensive knowledge of herbal use, which is essential for providing health-promoting services to the community [28].

### Data collection

The research team reported to the local council leaders of the study areas, who helped identify key TMP informants in their respective areas. When the gatekeepers failed to identify all the TMPs in their areas, snowball sampling was used. An ethnobotanical survey was carried out among TMPs using a pilot-tested semi-structured questionnaire to gather comprehensive information on the use of medicinal plants and other materials for malaria prevention. All data were collected in the common local languages spoken in the study areas (Adjumani District: Madi; Madi-Okollo District: Madi/Lugbara/Jonam/Alur; Moyo District: Madi; Obongi District: Madi/Lugbara; Pakwach District: Alur/Jonam) with the help of trained research assistants familiar with the study areas and local languages. The questionnaire was designed to collect information such as the study locality, respondents’ socio-demographic details (age, gender, education level, and occupation), plant names, parts used, harvesting methods, plant sources, preparation, and administration methods.

### Plant collection and identification

The plants were collected following the World Health Organization’s guidelines on good agricultural and collection practices (GACPs) for medicinal plants [29]. Voucher specimens of all mentioned plant species were collected and prepared according to Martin [30] and then deposited at the Makerere University Herbarium for identification, classification, and storage for future reference. The voucher specimens were further verified using POWO, which was accessed online at https://powo.science.kew.org.

### Data analyses

All the data were analyzed using Microsoft Excel version 16.53, 2021. Descriptive statistics, such as frequencies and percentages, were used to present respondents’ socio-demographic details and knowledge of mosquito repellent plant species in tables, bar graphs, and pie charts.

## Results

**Fig. 2:**
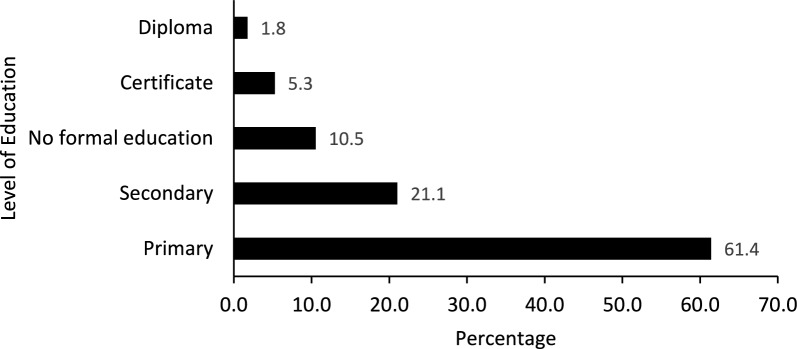
Level of education

Fifty-seven respondents were interviewed, 6 from Adjumani, 12 from Moyo, 17 from Pakwach, 12 from Madi-Okollo, and 10 from Obongi districts. Most of the respondents (44: 77.2%) were male, while the rest were female. The average age of the respondents was 57.6 years. In addition, the majority of the respondents (61.4%) had the Primary level of education (Fig. 2). Nearly all the respondents (89.5%) were primarily peasant farmers, with the exception of one mechanic (1.8%), one carpenter (1.8%), and four teachers (7.8%).

Forty-two plant species from 40 genera and 25 families were recorded as being used for repelling mosquitoes (Table 1). The most commonly used plants for repelling mosquitoes were: *Azadirachta indica *A.Juss. (9)*, Boswellia papyrifera* (Caill.) (7), *Aeschynomene american*a L. (6), *Mesosphaerum suaveolens* (L.) Kuntze. (6), and *Ocimum gratissimum* L. (5).

**Table 1:** Plant species used as mosquito repellents in the West Nile Subregion

Family, scientific name and voucher number	Local name (language)	Habit	Part used	Method of preparation and administration	FoM
Acanthaceae					
1. *Nicoteba betonica* (L.) Lindau (Syn: *Justicia betonica* L.) (AG 1100)	Okangali/mawua (Madi)	H	L & St	Burn to smoke the house	1
Asteraceae					
2. *Tagetes minuta* L. (AG 1114)		H	L	Burn to smoke the house	1
Bignoniaceae					
3. *Stereospermum kunthianum* Cham (AG 1115)	Liyo/lio (Madi)	Sh	R	Burn to smoke the house	2
Boraginaceae					
4. *Trichodesma zeylanicum* (Burm.f) R.Br (AG 1113)	Anyakanya/Ewanyaku	H	Wp	Burn to smoke the house	1
Burseraceae					
5. *Boswellia papyrifera* (Caill.) Hochst (AG 1116)	Beiswe (Madi)	H	Re	Burn to smoke the house	7
Capparaceae					
6. *Crateva adansonii* DC (AG 1139)	Eleu (Madi)	T	Wp	Burn to smoke the house	1
Chenopodiaceae					
7. *Chenopodium opulifolium* Schrad. ex W.D.J.Koch & Ziz (AG 1112)	Njai agori (Madi)	H	R	Burn to smoke the house	3
Cleomaceae					
8. *Cleome gynandra* L. (AG 1128)	Akeyo (Alur/Jonam)	H	Wp	Burn to smoke the house	3
Combretaceae					
9. *Combretum molle* G. Don (AG 1110)	Mei bi/Imeiku (Madi)	T	St & L	Burn fresh plant to smoke the house	1
Cucurbitaceae					
10. *Cucumis melo* L. (Syn: *Luffa cylindrica* (L.) M.Roem.) (AG 1117)	Lipa/changwa	H	Wp	Burn to smoke the house	1
Euphorbiaceae					
11. *Manihot esculenta* Crantz (AG 1140)	Ongura (Alur/Jonam)	Sh	Tb	Burn to smoke the house	2
12. *Jatropha curcas *L. (AG 1109)	Olu/Wulu (Madi)	Sh	L & Sd	Burn fresh to smoke the house	2
Fabaceae					
13. *Aeschynomene american*a L. (AG 1138)	Nyoricingi (Madi)	H	L	Burn to smoke the house	6
14. *Afzelia africana* Sm.ex Pers (AG 1108)	Meli (Madi)	T	B	Burn to smoke the house	2
15. *Albizia coriaria* Welw. Ex Oliv (AG 1127)	Uber (Alur/Jonam)	T	B	Burn to smoke the house	2
16. *Daniellia oliveri* (Rolfe) Hutch. & Dalziel (AG 1137)	Bito/Bitoki (Madi)	T	Re	Burn to smoke the house	1
17. *Chamaecrista nigricans* (Vahl) (AG 1107)	Liro eti/Iliro eti (Madi)	T	R	Burn to smoke the house	1
18. *Entada wahlbergii* Harv. (AG 1136)	Gala mombe (Madi)	T	B	Burn to smoke the house	1
19. *Philenoptera laxiflora* (Guill. & Perr.) Roberty (AG 1118)	Lokobe (Madi)	T	L	Burn to smoke the house	1
20. *Senna obtusifolia* (L.) H.S. Irwin & Barneby (AG 1106)	Kilikili (Lugbara)	Sh	L	Burn to smoke the house	1
21. *Vachellia seyal* (Delile) P.J.H. Hurter (AG 1126)	Aligo oci/ali (Madi)	T	St/L	Burn fresh plant to smoke the house	1
22. *Vachellia sieberiana* (DC.) Kyal. & Boatwr. (AG 1135)	Asoro (Madi)	T	R	Burn fresh plant to smoke the house	1
Geraniaceae					
23. *Pelargonium alchemilloides* (L.) L’Hér (AG 1105)		H	L	Burn fresh plant to smoke the house	1
Lamiaceae					
24. *Hoslundia opposita* Vahl (AG 1134)	Vungelee (Jonam)	H	L	Burn to smoke the house	1
25. *Mesosphaerum suaveolens* (L.) Kuntze (AG 1119)	Ajiri (Madi)	H	L	Plant around the compound/Burn to smoke the house	6
26. *Plectranthus* spp. (AG 1104)		H	L	Burn to smoke the house	1
27. *Ocimum americanum* L. (AG 1129)	Adiradira (Madi)	H	L	Place around the bed/burn fresh to smoke the house	3
28. *Ocimum gratissimum* L. (AG 1120)	Anguliel (Alur/Jonam)	H	Wp	Burn to smoke the house	5
Malvaceae					
29. *Gossypium hirsutum* L. (AG 1141)	Pamba (Alur/Jonam)	Sh	Fr/Sd/Wl	Burn to smoke the house	2
Meliaceae					
30. *Azadirachta indica* A.Juss (AG 1122)	Neem (Alur/Jonam)	T	L	Burn to smoke the house	9
31. *Melia azedarach* L (AG 1133)	Lira (Madi)	T	L & Sd	Burn to smoke the house	1
Moraceae					
32. *Antiaris toxicaria* (J.F.Gmel.) Lesch (AG 1132)	Odo/Ripi (Madi)	T	L	Burn fresh leaves to smoke the house	1
Moringaceae					
33. *Moringa oleifera* Lam (AG 1121)	Tangu/Moringa (Madi/Jonam)	T	R	Burn to smoke the house	1
Musaceae					
34. Musa spp. (AG 1101)		H	L	Burn to smoke the house	1
Myrtaceae					
35. *Eucalyptus* spp. (AG 1124)	Kalafuru (Alur/Jonam)	T	L	Burn to smoke the house	2
Poaceae					
36. *Cymbopogon citratus* (DC.) Stapf (AG 1123)	Lum chai (Alur/Jonam)	H	Wp	Planting in the compound	4
37. *Eleusine coracana* (L.) Gaertn (AG 1125)		H	Hk	Burn to smoke the house	1
Polygalaceae					
38. *Securidaca longepedunculata* Fresen (AG 1142)	Oyoefi/Oyorofifi (Madi)	Sh	St & L		
Polygonaceae					
39. *Oxygonum sinuatum* (Meisn.) Dammer (AG 1130)	Orogo ociki (Madi)	H	L	Burning to smoke the house	1
Sapotaceae					
40. *Vitellaria paradoxa* C.F.Gaertn (AG 1102)	Awa ekwi (Madi)	T	Sd	Burn seed residue to smoke house/ mix with red algae, and smear on skin	2
Solanaceae					
41. *Solanum incanum* L. (AG 1131)	Kilinga/Olilinga (Lugbara)	H	L	Burn to smoke the house	1
Zygophyllaceae					
42. *Balanites aegyptiaca *(L.) Delile (AG 1103)	Thoo (Alur/Jonam)	T	Sd coat	Pound and burn seed coat	2

*B* bark, *FoM* frequency of mention, *Fr* flower, *H* herb, *Hs* husk, *L* leaves, *R* root, *Re* resin, *Sd* seed, *Sh* shrub, *St* stem, *T* tree, *Tb* tuber, *Wl* wool, *Wp* whole plant

**Fig. 3:**
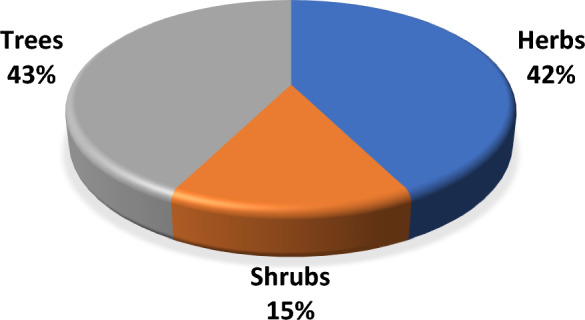
Life forms of the plant species used to repel mosquitoes in the West Nile Subregion

Most of the plant species used were trees (43%) or herbs (42%) (Fig. 3).

**Fig. 4:**
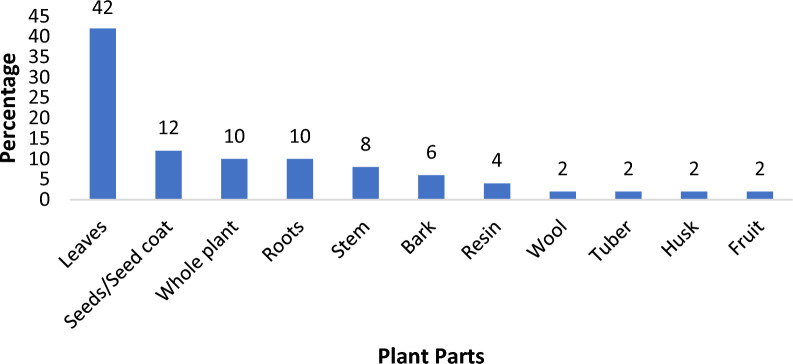
Parts of the plant species used to repel mosquitoes in the West Nile Subregion

The most commonly used plant parts were the leaves (42%) and seeds (12%) (Fig. 4).

**Fig. 5:**
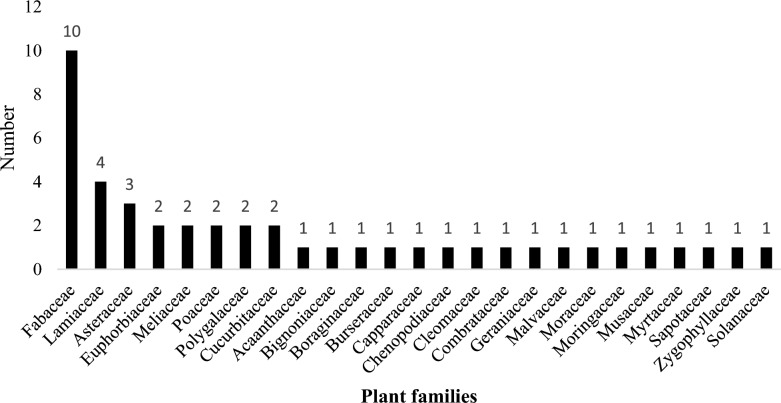
Plant families

Most of the plant species were from the Families Fabaceae (10), followed by Lamiaceae (4) and Asteraceae (3) (Fig. 5).

### Methods of preparation and and drug administration

**Fig. 6:**
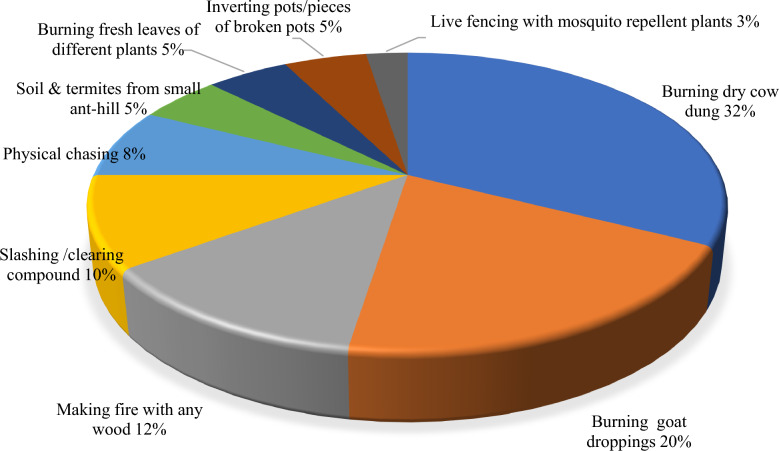
Other indigenous methods for repelling mosquitoes

The preparation of all the plant species used, except *Cymbopogon citratus,* involved burning them to smoke the house. Both *Cymbopogon citratus* and *Mesosphaerum suaveolens* were planted around homesteads or houses to repel mosquitoes. The latter was used as both a live hedge and burnt to repel mosquitoes. In addition to the use of plants, the other common indigenous methods for repelling mosquitoes involved burning either dry cow dung (32%) or dry goat droppings (20%), making fire with any wood (12%), or slashing/clearing the compound (10%) (Fig. 6).

## Discussion

### Documented plants with mosquito repellent properties

Although several plant species with insect-repelling properties exist in the tropics [21], they have mostly remained unexplored, with only a few studies documenting the medicinal plant species used as repellents in Africa in general and in Uganda specifically. Limited documentation has prevented most of these plant species from being scientifically evaluated for their effectiveness.

Compared with our study, where 42 plant species were documented as being used traditionally for repelling mosquitoes in the West Nile Subregion, Uganda, Pavela and Benelli [31] conducted a review of plants used to repel mosquitoes across Africa and reported that natives in Africa (Ethiopia, Kenya, Nigeria, South Africa, and Tanzania) traditionally used 64 plants from 30 families. In addition, six plant species specifically used for repelling mosquitoes were previously documented in Cegere, northern Uganda [32]. Similarly, Shibeshi et al. [20] documented 19 plant species used by the local community as mosquito repellents in Seweyna district, Ethiopia, whereas Mavundza et al. [33] documented 23 plant species used in the uMkhanyakude district of KwaZulu-Natal province, South Africa. Furthermore, Pålsson and Jaenson [34] documented eight plant species used for repelling mosquitoes in the Oio region of Guinea-Bissau. Seyoum et al. [35] reported eight plant species from Kenya, and Havyarimana et al. [36] reported 12 from Burundi. Kweka et al. [37] reported essential oils from two plant species, *Ocimum suave* and *Ocimum kilimandscharicum*, from Northeast Tanzania, which are used as mosquito repellents.

The most commonly used plants for repelling mosquitoes in our study were *Azadirachta indica *A.*, Boswellia papyrifera* (Caill.), *Aeschynomene american*a L., *Mesosphaerum suaveolens* (L.) Kuntze (Syn: *Hyptis suaeolens*), and *Ocimum gratissimum* L. These plants contain phytochemicals that exhibit mosquito-repelling properties. For example, two different Boswellia species, *Boswellia microphylla* and *Boswellia neglecta*, are among the most commonly used mosquito repellent plants in Ethiopia [20]. The same Boswellia species documented in our study (*Boswellia papyrifera*) was also used by the Oromo people in Ethiopia [38]. Another interesting overlap was the use of a different species of Ocimum (*Ocimum ellenbeckii*) and two different Acacia species (now *Vachellia*), *Acacia mellifera* Benth and A*cacia bussei* Harms.ex. Joste in Ethiopia, whereas *V. seyal* and *V. siberiana* were documented in this study*.*

In Kenya, other than *L. camara*, the other commonly documented mosquito repellent medicinal plant species (*Ocimum americanum* L*.*, *Tagetes minuta*, *A. indica*, and *H. suaveolens*) [35] or members of the same genus (O*. americanum*) were also used as mosquito repellents in this study. In addition, *M. azedarach* as well as *Balanites maughamii* Sprague and *Balanites aegyptiaca,* were documented in South Africa as mosquito repellents [33]*,* and *M. azedarach* was used among the Oromo people in Ethiopia [38]. The similarity in usage across countries supports the ethnobotanical use of these medicinal plants for mosquito repellency by the West Nile community in Uganda.

Similarly, in Guinea-Bissau, West Africa, Pålsson and Jaenson [34] documented some of the most commonly used mosquito repellent plant species, such as *Hyptis suaveolens* Syn: *Mesosphaerum suaveolens, Daniellia olieri*, *Elaeis guineensis* Jacq., *Parkia biglobosa* (Jacq. Benth.), *Azadirachta indica*, *Eucalyptus* sp., *Ocimum canum* Sims, and *Senna occidentalis* (L.) Link. Interestingly, except for *Parkia biglobosa,* all the other identified plant species or their genera are also frequently used in our study area as mosquito repellents (*O. canum* and *S. occidentalis*).

### Methods of preparation and drug administration

The methods of preparation and/or use of the mosquito repellent plant species recorded in this study are similar to those reported in previous studies. The most typical traditional method of preparation and administration for plants is the burning of the materials to produce smoke indoors. Similarly, in northern Uganda, the same practice was reported, with many plant species used as mosquito repellents. For example, the peels of *Musa* spp., *Ocimum forsskaolii,* and *Manihot esculenta* are sun-dried and burned inside a house to produce smoke that repels mosquitoes [32]. The same practice was also reported in Guinea-Bissau [34] and in Ethiopia among the Oromo ethnic group, where, for example, *C. citratus* is prepared by smoking [38]. The widespread use of the traditional method, which involves burning plant parts to smoke the houses to repel mosquitoes, could be linked to the volatile nature of the essential oils found in the various parts of the plants used. As the parts burn, the essential oils evaporate into the smoke and come into contact with the mosquitoes flying in the surrounding area, killing or repelling them. Moreover, some of the plants documented in this study, such as *C. citratus*, were freshly prepared. In Guinea-Bissau, West Africa, mosquito repellents are also used fresh, probably because of the volatile phytochemicals they contain [34]. In contrast, both *C. citratus* and *M. suaveolens* were also planted around homesteads or houses to repel mosquitoes [34].

Remarkably, the most popular methods that do not involve directly using plants to repel mosquitoes include burning either dry cow dung or dry goat droppings to produce smoke. This is because smoke from cow dung contains mixtures of chemicals that irritate or block the sensory receptors of the mosquitoes [39]. This practice has also been reported in India [40] and parts of Ethiopia [41].

### Efficacy of the plants used

Some of the plant species used as repellents are aromatic in nature, meaning that they release essential oils. These natural mosquito repellents could help address the high levels of insecticide resistance [19]. Plant-derived essential oils and extracts can be used in the formulation of environmentally friendly repellents. Plant-based oils are often effective alternatives to synthetic repellents because they are generally safe, cost effective, and widely available in many regions worldwide [19]. Several studies have shown the effectiveness of essential oils from various plants as mosquito repellents. For example, oils from citronella, clove, eucalyptus, geranium, lavender, peppermint, and other species have been tested for repellency in the laboratory against *Aedes aegypti* [42] and other species, confirming their efficacy [21]. However, Maia et al. [43] concluded in a Cochrane review that there is insufficient evidence to determine whether topical or spatial repellents can prevent malaria.

## Conclusion

The communities in the West Nile Subregion, especially those living along the River Nile, use a rich diversity of plant species to repel mosquitoes. Many plant species used as mosquito or insect repellents in the West Nile Subregion are also widely used as repellents in other African countries. The most commonly used plants for repelling mosquitoes include* A. indica, B. papyrifera*, *A. american*a, *M. suaveolens*, and *O. gratissimum*. Most of the plant species used are burned to produce smoke. Burning cow dung or goat droppings was also used to repel mosquitoes. We recommend further research into the mosquito-repelling properties of these plants to explore their potential for the development of alternative mosquito repellent products**.**

## Data Availability

No datasets were generated or analyzed during the current study.

## Abbreviations

GACP: Good agricultural and collection practices
POWO: Plants of the world online
TMP: Traditional medicine practitioner
WHO: World Health Organization
